# Bioaggregachromism of Asymmetric Monomethine Cyanine Dyes as Noncovalent Binders for Nucleic Acids

**DOI:** 10.3390/bios15030187

**Published:** 2025-03-14

**Authors:** Sonia Ilieva, Nikolay Petkov, Raimundo Gargallo, Christo Novakov, Miroslav Rangelov, Nadezhda Todorova, Aleksey Vasilev, Diana Cheshmedzhieva

**Affiliations:** 1 Faculty of Chemistry and Pharmacy, Sofia University “St. Kliment Ohridski”, 1 J. Bourchier Ave., 1164 Sofia, Bulgaria; silieva@chem.uni-sofia.bg (S.I.); ahnp@chem.uni-sofia.bg (N.P.); 2 Department of Chemical Engineering and Analytical Chemistry, University of Barcelona, Martí i Franqués 1-11, E-08028 Barcelona, Spain; raimon_gargallo@ub.edu; 3 Institute of Polymers, Bulgarian Academy of Sciences, Akad. G. Bonchev Str., Bl.103A, 1113 Sofia, Bulgaria; hnovakov@polymer.bas.bg; 4 Institute of Organic Chemistry with Centre of Phytochemistry, Bulgarian Academy of Sciences, 1113 Sofia, Bulgaria; miroslav.rangelov@orgchm.bas.bg; 5 Institute of Biodiversity and Ecosystem Research, Bulgarian Academy of Sciences, 1113 Sofia, Bulgaria; nadeshdahr@gmail.com

**Keywords:** aggregachromism, cyanine dyes, nucleic acids, fluorogenic probes, molecular recognition, bioaggregachromism

## Abstract

Two new asymmetric monomethine cyanine dyes, featuring dimethoxy quinolinium or methyl quinolinium end groups and benzothiazole or methyl benzothiazole end groups were synthesized. The chemical structures of the two dyes—(*E*)-6,7-dimethoxy-1-methyl-4-((3-methylbenzo[d]thiazol-2(3H)-ylidene)methyl)quinolin-1-ium iodide (**3a**) and (*E*)-4-((3,5-dimethylbenzo[d]thiazol-2(3H)-ylidene)methyl)-1,2-dimethylquinolin-1-ium iodide (**3b**)—were confirmed through NMR spectroscopy and MALDI-TOF mass spectrometry. A new methodology was developed to study monocationic dyes in the absence of a matrix and cationizing compounds in MALDI-TOF mass experiments. The newly synthesized dyes contain hydrophobic functional groups attached to the chromophore, enhancing their affinity for the hydrophobic regions of nucleic acids within the biological matrix. The dyes’ photophysical properties were investigated in aqueous solutions and DMSO, as well as in the presence of nucleic acids. The dyes exhibit notable aggregachromism in both pure aqueous and buffered solutions. The observed aggregation phenomena were further elucidated using computational methods. Fluorescence titration experiments revealed that upon contact with nucleic acids, the dyes exhibit bioaggregachromism–aggregachromism on the surfaces of the respective biomolecular matrix (RNA or DNA). This bioaggregachromism was further confirmed by CD spectroscopy. Given the pronounced aggregachromism detected, we conclude that the dyes investigated in this study are highly suitable for use as fluorogenic probes in biomolecular recognition techniques. The unique absorption and fluorescence spectra of these dyes make them promising fluorogenic markers for various bioanalytical methods related to biomolecular recognition.

## 1. Introduction

Biomolecular probes are complex chemical compounds capable of labeling a specific type of biological macromolecules or target cell organelles [1,2,3]. After the interaction with the target bio objects, the molecular probes undergo a noticeable change in their photophysical properties [1,2,3]. A most desired phenomenon in biomedical assays is the appearance of a unique and clearly distinguishable signal [4]. In other words, biomolecular probes should be characterized by drastically low intrinsic fluorescence in the free state in solutions and acquire intense fluorescence after interaction with the target bio-objects [3,4,5]. In connection with the above, polymethine or so-called cyanine dyes are the most suitable for the mentioned purposes. This class of dyes is divided into several subgroups [2,3]. The most widely used types of structures are: monomethine cyanine dyes, which are used to label nucleic acids and are one of the major components of the PCR kits [1]. Additionally, polymethine cyanines— including tri-, penta-, hepta-, and nonamethine cyanines—are applied as fluorogenic markers for proteins and nucleic acids [3,6]. There is another unique feature in the photophysics of this class of dyes, which is their ability to aggregate in solution or on a specific surface, significantly changing their photophysical properties after aggregation [7]. This phenomenon is called aggregachromism. In the case of cyanine dyes, aggregachromism is highly pronounced and leads to disappearance or in some cases appearance of absorption and fluorescence bands [7].

The purpose of this article is to investigate and demonstrate the change in photophysical properties depending on the concentration of nucleic acids of two new asymmetric monocationic monomethine cyanine dyes. To accurately interpret and explain the obtained results and in support of experimental research, we use theoretical DFT and TDDFT computations to predict the types of aggregation architectures formed under the given conditions.

## 2. Results

### 2.1. Synthesis

**Scheme 1:**
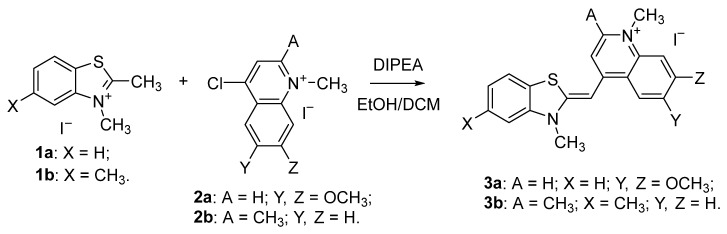
Synthesis of asymmetric monomethine cyanine dyes **3a** and **3b**.

Reaction conditions for the synthesis of the intermediates **1a**, **1b**, **2a**, and **2b** (Scheme 1) are described in the publication by Kandinska et al. [8]. The target dyes were obtained by grinding in a mortar the corresponding 2-methylbenzothiazolium salts 2,3-dimethylbenzo[d]thiazol-3-ium iodide **1a** or 2,3,5-trimethylbenzo[*d*]thiazol-3-ium iodide **1b** and a 20% molar excess of the 4-chloroquinolinium analogues 4-chloro-6,7-dimethoxy-1-methylquinolin-1-ium iodide **2a** or 4-chloro-1,2-dimethylquinolin-1-ium iodide **2b**. The fine grounded reaction mixture was transferred to a reaction flask and suspended in a mixture of equivalent amounts of dichloromethane and ethanol. Thus, in an inert atmosphere, with intensive stirring, we added dropwise the sterically hindered Huenig’s base—N,N-diisopropylethylamine.

The target dyes, which formed as orange (**3a**) and red (**3b**) precipitates, were isolated and purified through recrystallization from a mixture of ethanol and DCM. The chemical structures of the final compounds **3a** and **3b** were proven by NMR spectroscopy and MALDI-TOF spectrometry.

In the proton NMR spectra of dye **3a** (Figures S1–S5a–d), the protons for the methyl group attached to the nitrogen atom of the benzothiazole ring appear as a singlet with integral intensity for three protons at 3.94 ppm (Figure S5a). A second singlet with an intensity corresponding to six protons is located at 4.08 ppm. The third singlet is located at 4.22 ppm, with an integral intensity equal to three protons (Figure S5a). The latter singlet appears downfield, which is a sign that the methyl group corresponding to the singlet is connected to a strong electron-accepting center–the quaternary nitrogen atom of the quinolinium cycle. We attributed the singlet at 6.77 ppm (Figure S5b) with integral intensity corresponding to one proton of the monomethine group located between the two heterocycles of the chromophore (proton number 6). The signal at 7.34 ppm appearing as a doublet of doublets with a J constant of 8 Hz can be attributed to proton number 3 in the benzothiazole ring (Figure S5b). The singlet at 7.36 ppm (Figure S5b), which overlaps with the mentioned doublet of doublets, can be attributed to proton number 7 in the quinolinium heterocycle. The doublet with an integral for one proton at 7.42 ppm, with a J constant of 8 Hz, corresponds to proton number 5 in the benzothiazole moiety (Figure S5c). The next doublet of doublets at 7.55 ppm (Figure S5c) with an integral for one proton, J constant of 8 Hz, is assigned to proton number 4 in the benzothiazole moiety. The doublet at 7.68 ppm with a J constant of 8 Hz corresponds to proton number 2 of the benzothiazole fragment (Figure S5c). The singlet at 7.79 ppm most likely corresponds to proton number 8 of the quinolinium fragment (Figure S5c). The two doublets at 7.95 and 8.54 ppm, with J constants of 8 Hz, correspond to protons 10 and 9, respectively, located next to the quaternary nitrogen in the quinolinium heterocycle (Figure S5c). Considering the carbon spectra with those from the DEPT method and the HSQC method also fully supports the proposed structure of dye **3a** (Figures S5e,f–S7). In the ^13^C NMR (Figure S6), the number of carbon atoms is exactly as predicted. The comparison between the ^13^C NMR and the DEPT spectrum gives the exact number of quaternary carbon atoms. The number of methyl and methine groups also matches the proposed structure (Figures S5e,f–S7, Experimental part). The location of the signals in the NMR spectra of the other new analog-dye **3b** is quite similar. They are described in detail in Section 3 (Figures S8–S12). The number of signals matches the proposed chemical structure.

As an additional confirmation of the chemical structures of the obtained two new compounds, we also applied MALDI–TOF mass spectra of the two new dyes (Figures S13 and S14).

Usually, in this method, when studying the structure and purity of similar dyes, matrices such as ethanolamine and 2,2′-(1,4-phenylenebis(methaneyl-ylidene)dimalononitrile are used [9]. MALDI-TOF measurements of cationic or anionic dyes are usually held with the addition of a matrix to the analyzed sample that often leads to very strong electrostatic interactions or π–π-stacking of the dye molecules on the surface of the matrix, which interferes with the evaporation of the samples and does not lead to a precise result. Our attempts to add a matrix component into the sample solution did not give a good MALDI mass spectrum, probably due to self-aggregation of the dye molecules or aggregation on the matrix surface but not to their co-crystallization. Applying direct irradiation of the samples dropped on the plate without a matrix and salt cationizing compound and their subsequent volatilization led to very accurate results regarding the molecular mass peaks of the two new dyes **3a** and **3b** (Figures S13 and S14). Figure S13 shows the appearance of several satellite signals to the right of the molecular peaks that are exactly one unit apart from each other. This is due to the presence of sulfur isotopes (^32^S (95.02%), ^33^S (0.75%), ^34^S (4.21%), and ^36^S (0.02%)) in the benzothiazole heterocyclic moiety of the dye chromophores (Figure S15).

The methodology described for MALDI-TOF analysis is novel for this class of dyes and, to the best of our knowledge, has not yet been reported in the scientific literature. The cationic dyes are important for medical diagnostics and therefore thorough proof of their structure is required. That makes MALDI-TOF mass spectrometry, along with NMR analysis, an indispensable tool for characterizing the structure of newly synthesized compounds.

### 2.2. Photophysical Properties of the Dyes and Their Complexes with Nucleic Acids

#### 2.2.1. Aggregation Studies

**Figure 1:**
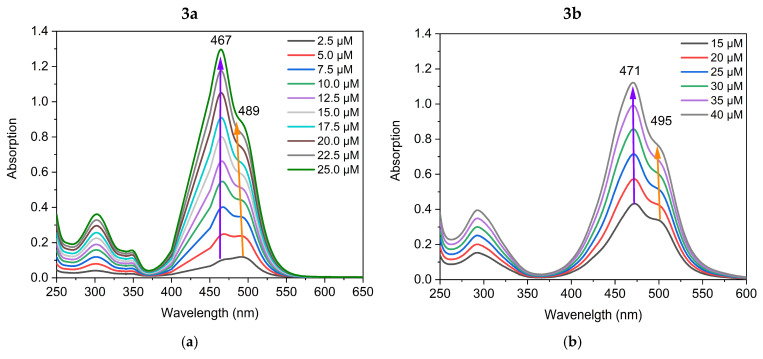
UV/Vis absorption spectra of solutions with increasing concentrations of dyes **3a** (**a**), and **3b** (**b**) in TE buffer at pH 8.

The visible light absorption of the dyes was characterized in TE buffer at pH 8. Two distinct absorption bands within the visible range of 400–550 nm were observed (Figure 1).

Dye **3a** is characterized by two overlapping absorption bands with maxima at 467 nm (50,331 L·mol^−1^·cm^−1^) and 489 nm (30,671 L·mol^−1^·cm^−1^) (Figure 1). At low concentrations of dye **3a**, the maximum at 467 nm has lower intensity. In a starting concentration at 2.5 µM solution of **3a**, the most intensive peak is at 489 nm (Figure 1). When the dye concentration in TE buffer increases, the maximum at 467 nm significantly increases in intensity, becoming the most intensive peak at a concentration of 25 µM. Based on this observation, we assigned the 467 nm maximum to the aggregated form of the dye. The absorption spectrum of the compound **3b** in the visible range 400–600 nm is similar to that of **3a** and shows two overlapping bands at 471 nm (28,080 L·mol^−1^·cm^−1^) and 495 nm (17,380 L·mol^−1^·cm^−1^), Figure 1. Dye **3b**, in contrast to **3a**, exhibits a dominant shorter wavelength maximum at 471 nm even at low concentrations (Figure 1). This behavior could be attributed to the higher hydrophobicity of dye **3b** compared to dye **3a** that results in stronger aggregation. Cyanine dyes tend to aggregate in aqueous solution due to their hydrophobicity and polarizability, which favor π-stacking interactions [10]. Biver et al. reported that self-aggregation of both Thiazole orange and its analogues occurs in different strengths depending on the dye itself [11]. The aggregation processes of monomethine cyanine dyes have also been recorded in our previous studies [8,12]. However, for the first time, we establish the formation of extremely stable molecular aggregates. It was of significant interest to explore the influence of the formation of stable aggregates on the dye-NA interactions.

**Figure 2:**
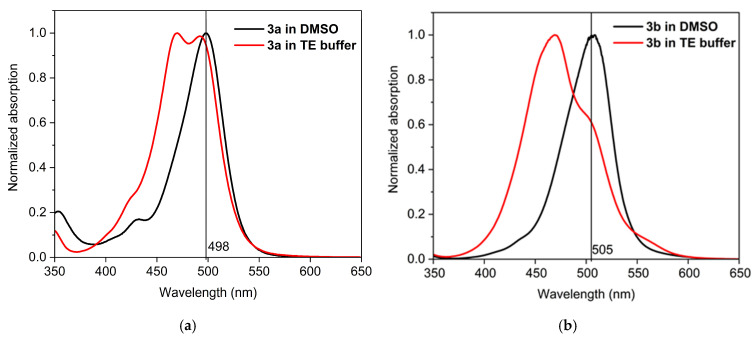
UV/Vis spectra of the dyes **3a** (**a**) and **3b** (**b**) in DMSO and in TE buffer.

To investigate and demonstrate the formation of aggregates in TE buffer and to distinguish between the monomer and aggregate bands, the UV/Vis spectra of the dyes were recorded in both DMSO and TE buffer solution. The UV/Vis spectra of the dyes in DMSO and in TE buffer are compared in Figure 2. The novel cyanine dyes dissolve readily in DMSO and show no tendency to aggregate in this solvent over time. It is worth mentioning that these monomethine cyanine dyes are not solvatochromic, as far as the bands’ maxima do not differ significantly in DMSO and water, as can be seen from Figure 2. This suggests that the differences in DMSO and TE spectra are due to aggregation rather than solvatochromism or acidochromism. In DMSO, the dyes exist only as monomers, exhibiting a strong absorption around 500 nm (Figure 2): the dye **3a** shows only one band at 498 nm associated with the monomeric form (Figure 2a) and the dye **3b** exhibits only a monomeric band at 507 nm (Figure 2b). The band at 464 nm in TE buffer (Figure 2a) and 478 nm (Figure 2b) is associated with the self-aggregation of dyes **3a** and **3b**, respectively. In a water medium, the dyes form associates characterized with shorter wavelength absorption bands, while the monomer bands show lower intensity (Figure 2).

Theoretical modeling of the aggregation process was applied to gain a deeper understanding of the aggregation behavior.

#### 2.2.2. Quantum Chemical Calculations

Quantum chemical computations were applied to determine the geometry, electronic structure, and spectral properties of the studied cyanine dyes. The geometry optimization and photophysical properties of the dyes’ monomers and dimers were computed using the G16 software package [13]. Full geometry optimization at DFT level M062X/6-31G(d,p) [14] in water was performed. All computations were performed in a water medium to reproduce the experimental conditions (TE buffer). The effect of the medium was taken into account by means of PCM formalism [15,16]. To verify that each optimized structure is a minimum on the potential energy surface, an analysis of the harmonic vibrational frequencies was performed using the same method/basis set, and no imaginary frequency was found. The M062X functional was specifically chosen for the optimization of the dimers and in order to maintain π-stacking [17]. The absorption maxima of each monomer and dimer were calculated at HFS/6-31+G(d,p) [18,19] level of theory, which was found to be among the most effective in predicting absorption and emission transitions for cyanine dyes [17].

**Figure 3:**
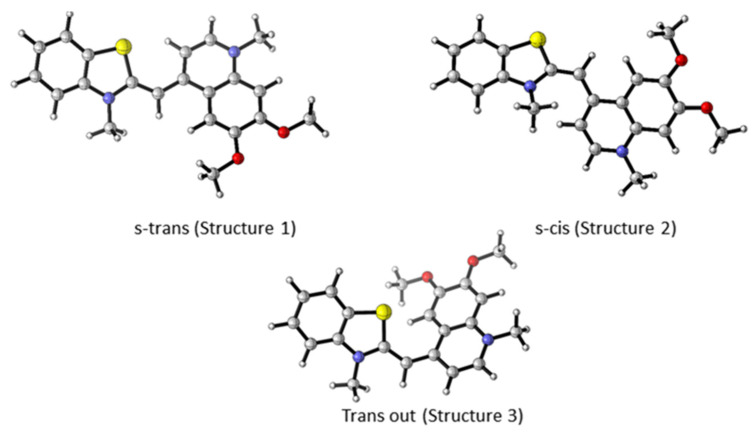
M062X/6-31G(d,p) optimized geometry of different conformers of **3a**. Color scheme: O—red, N—blue, S—yellow, C—gray, H—light gray [20].

For each dye, three main conformers were studied (Figure 3). The main difference between the studied conformers is the torsion S-C-C-H and the dihedral angle between the two linked heterocycles, benzothiazole and quinoline. The optimized geometries of each conformer of dye **3a** are presented in Figure 3. The most stable conformer, Structure 1 (s-trans), is characterized by the two heterocycles lying in the same plane. Structure 2, referred to as s-cis, is sterically more restricted, as shown in Figure 3. The s-trans conformer is more stable than the s-cis conformer. The free energy difference between the s-cis and s-trans conformers is 3.4 kcal/mol and 3.6 kcal/mol in favor of the s-trans conformer for **3a** and **3b**, respectively (Table 1).

The computed atomic charges (NBO, MK, and Hirschfeld) for the three conformers of dyes **3a** and **3b**, along with the electrostatic potential at the nuclei (EPN), are summarized in Table 2. Hirshfeld charges are more effective in describing the properties of aromatic molecules [21]. The nitrogen atom N2 (quinoline moiety) is positively charged in all three forms, as expected. The benzothiazole nitrogen atom, N1, is positively charged only in the cis form of dye **3b**. EPN values accurately reflect changes in electron density without additional approximations that are intrinsic to atomic charges depending on their definition. The more negative the EPN value, the more negatively charged the atom. The trend in the calculated EPN values corresponds to Hirshfeld charges. The correlation coefficient between Hirshfeld charges and EPN is 0.985.

**Figure 4:**
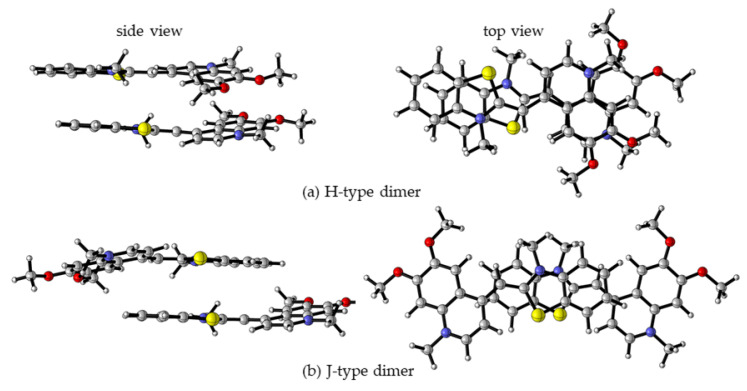
M062X/6-31G(d,p) optimized molecular structures of **3a** H- (**a**) and J-dimers (**b**) in a water medium: left—side view, right—top view. Color scheme: O—red, N—blue, S—yellow, C—gray, H—light gray [20].

As mentioned previously, monomethine cyanine dyes tend to aggregate in aqueous solution due to hydrophobic interactions. It is suggested that the hydrophobicity and polarizability of dyes favor π-stacking interactions in dimers and aggregates. The UV/Vis spectra of the dyes reveal a pronounced tendency for molecular aggregation. The structure of potential dimers has been examined theoretically, and the results are compared with the experimental spectral data. Two general types of dimers are discussed in the literature–H- and J-dimers [10,22,23]. H-dimers are formed through interactions between two dye molecules superimposed on each other (Figure 4a), whereas J-dimers arise when the molecules slide against each other (Figure 4b). A total of eight dimers were optimized for each molecule–four π-stacked H-dimers and four J-dimers. The optimized geometries of the most stable H- and J- dimers for dye **3a** are shown in Figure 4.

The self-aggregation has been found to occur with different strengths depending not only on the environment but on the dye itself. The relative energies and the calculated Boltzmann distributions for each of the eight studied dimers of **3a** dye are given in Table 3. J2 dimer has not been optimized. Among all dimers, the most stable one is H2 (Figure 4a, Table 3).

The Gibbs free energy of dimer formation was calculated using the M062X/6-31G(d,p) method for the following model reaction: 2D → (D)_2_. The calculated Gibbs free energy of dimer formation for the H2 dimer of dye **3a** is −5.7 kcal/mol. This theoretical value, along with the previously discussed UV/Vis spectra, indicates that the new dyes are highly prone to strong aggregation. The absorption maxima for both monomers and the most stable dimers, calculated at the HFS/6-31+G(d,p) level, are summarized in Table 4. Theoretical calculations indicate that all dimers, regardless of their type, exhibit a lower absorption wavelength (hypsochromic shift) compared to the dye monomer. This finding supports the assignment of the experimental absorption bands.

The formation of H-type trimers was also modeled. The optimized geometry is shown in Figure S16. The computed absorption maximum is 474 nm (f = 1.6726) 2.6161 eV. The Gibbs free energy of trimer formation, calculated using the M062X/6-31G(d,p) method for the model reaction: 3D → (D)_3_, is −6.3 kcal/mol. The formation of trimers is possible; however, the complex between NA and the trimers is less plausible.

The molecular orbitals involved in the S_0_ → S_1_ transitions and their energies for the monomer, dimer, and trimer were calculated, and visualized in Figure S17. In the H2 dimer, the energy difference between the orbitals involved in the transition (HOMO-1 and LUMO+1) is higher than for the monomer, which explains the hypsochromic shift in the absorption maxima for dimer aggregates.

#### 2.2.3. UV/Vis Spectra of Dye-NA Complexes

**Figure 5:**
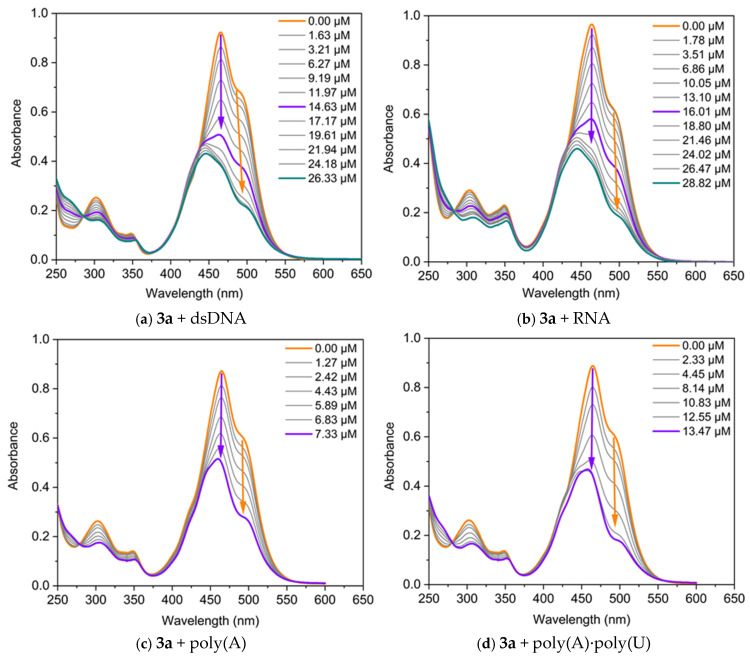

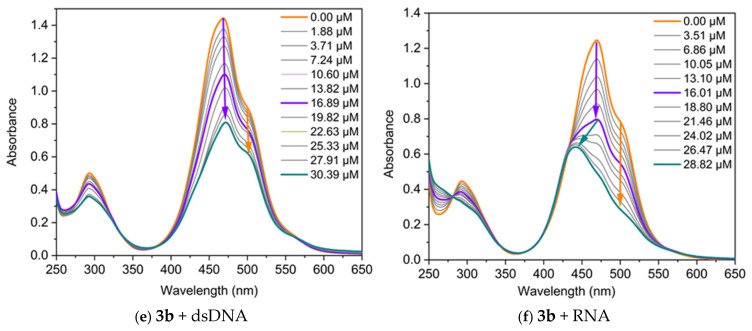
UV/Vis absorption of the dyes, neat and in the presence of increasing concentrations of nucleic acids, in TE buffer at pH 8: (**a**) **3a** C = 17.5 µM and dsDNA; (**b**) **3a** C = 17.5 µM and RNA; (**c**) **3a** C = 17 µM and poly(A); (**d**) **3a** C = 17 µM and poly(A)·poly(U); (**e**) **3b** C = 50 µM and dsDNA; (**f**) **3b** C = 45 µM and RNA. The arrows represent the direction of change in the absorbance of the dye-NA complexes as the nucleic acid concentration increases. The orange arrow indicates the decrease in intensity of the band associated with the monomer-NA complex, while the violet arrow denotes the change in the band related with the dye dimer-NA complex. The green arrow highlights the hypsochromic shift of the band.

The interaction of the dyes with nucleic acids (NA) was studied using UV/Vis and fluorescence spectroscopy. Spectrophotometric titrations were conducted by maintaining a fixed concentration of the dyes while varying the concentration of the polynucleotides. The changes in the absorption spectra of dyes **3a** and **3b** upon the addition of specific quantities of dsDNA, RNA, poly(A), and poly(A)·poly(U) are presented in Figure 5. Both dyes exhibit changes in their absorption spectra upon binding to nucleic acids, displaying pronounced hypochromism and, in some cases, bathochromic shifts.

The three main non-covalent binding modes of small molecules to nucleic acids are intercalation between base pairs, groove binding, and external electrostatic interactions. The intercalation of small organic molecules into DNA results in a bathochromic and hypochromic shift in the absorption spectrum of these molecules [24]. This bathochromic shift occurs because of a decrease in the π→π* transition energy, which arises from the coupling between the π bonding orbitals of the nucleic acid base pairs and the empty π* antibonding orbitals of the ligands. The bathochromic shift associated with intercalation is frequently greater than 15 nm [25,26]. In the case of groove-binding interactions, typical red shifts of less than 8 nm have been reported [27,28], which are due to the reduced direct contact between π-systems. Finally, external electrostatic interactions between the positively charged organic molecules and negatively charged nucleic acid backbone usually induce a hyperchromic effect [29].

It can be seen from Figure 5 that two main types of changes are observed in the titration curves: (1) both bands change synchronously, with their intensity decreasing as the nucleic acid concentration increases during titration; and (2) the longwave band (the monomeric one) decreases in intensity, while the shortwave band also diminishes in intensity up to a certain nucleic acid concentration, after which a hypsochromically shifted band emerges. Therefore, the dye molecules interact with nucleic acids, forming two types of complexes: monomer-NA and dimer-NA, as evidenced by the reduction in the intensity of both bands. In the case of dye **3a**, the band at 490 nm, associated with the monomeric dye, shifts to 504 nm in the final spectrum (∆λ = 14 nm), suggesting an intercalative mode of interaction for the monomer (Figure 5a). On the other hand, the band at 464 nm remains unchanged during titration with dsDNA, indicating a groove-binding mode of interaction for the dimers of **3a**. Based on this UV-Vis spectrum alone, a mixed type of interaction is expected. The effect of monomer–nucleic acid interactions on absorption spectra is typically referred to as biochromism. It is well known that in the cyanine class of dyes, the formation of aggregates can strongly influence the spectra, a phenomenon referred to as aggregachromism. In our case, the formation of aggregates on the nucleic acid matrices and the subsequent effect observed on the spectra can be termed bioaggregachromism.

The dye **3b** exhibits distinct titration profiles with dsDNA and RNA, reflecting differences in dye–nucleic acid interactions. When a dsDNA solution is added to **3b**, the intensity of both bands decreases, while their positions remain unchanged, further supporting a groove-binding mechanism [24,27]. In contrast, titration with RNA shows a different behavior, characterized by hypochromic and hypsochromic changes in the spectra (Figure 5f). This change is likely due to the formation of different complexes between dye dimer and the RNA.

#### 2.2.4. Fluorescent Properties of Dye-NA Complexes

**Figure 6:**
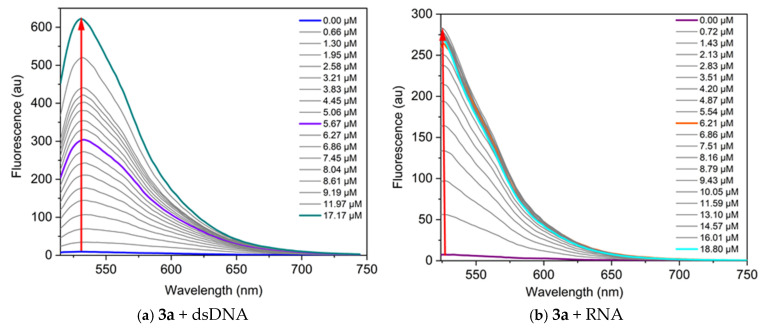

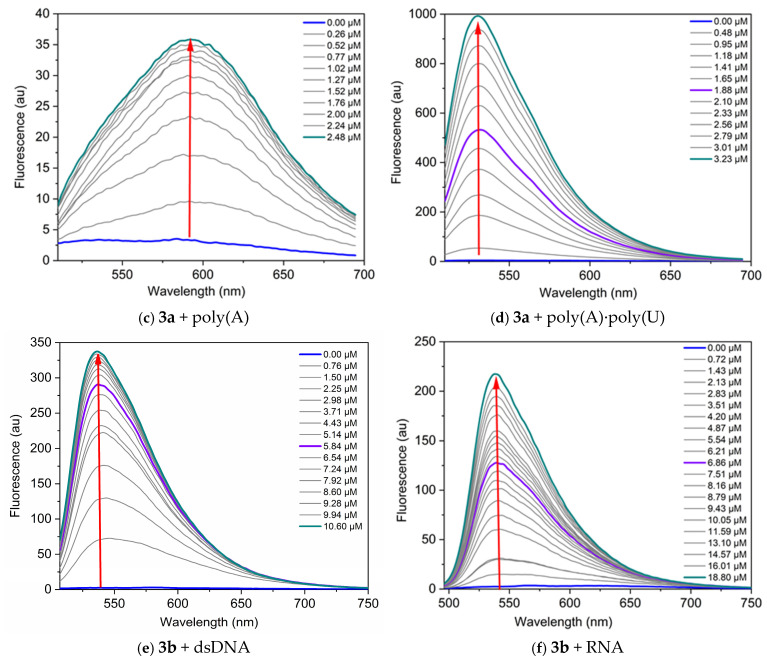
Fluorescence spectra of the dyes, neat and in the presence of increasing concentrations of nucleic acids, in TE buffer at pH 8: (**a**) **3a** C = 1 µM and dsDNA, λ_ex_ = 495 nm; (**b**) **3a** C = 1 µM and RNA, λ_ex_ = 504 nm; (**c**) **3a** C = 1 µM and poly(A), λ_ex_ = 497 nm; (**d**) **3a** C = 1 µM and poly(A)·poly(U), λ_ex_ = 497 nm; (**e**) **3b** C = 5 µM and dsDNA, λ_ex_ = 497 nm; (**f**) **3b** C = 1 µM and RNA, λ_ex_ = 476 nm. The red arrows indicate the change of fluorescence intensity as the nucleic acid concentration increases.

Additional information regarding the dye’s sensitivity and binding affinity is provided by the fluorescence response upon binding to nucleic acids. We examined the fluorescence of the dyes and their complexes with nucleic acids. In the fluorescence titration experiment, the dye concentration is kept constant (in the micromolar range), and a nucleic acid solution of specific concentration is gradually added to the dye solution, followed by a 5 min incubation before measuring fluorescence. Some representative results from the fluorescence titrations are shown in Figure 6. The dyes exhibit low intrinsic fluorescence in TE buffer; however, a significant increase in fluorescence intensity is observed upon binding to nucleic acids. The increase in fluorescence intensity after binding to nucleic acids indicates the interaction and formation of a non-covalent dye-nucleic acid complex.

Titration data were analyzed according to the site-independent model by nonlinear fitting [30]. The values of the binding constants K_b_ suggest strong interactions with the nucleic acids (Table 5).

The dyes studied demonstrate greater sensitivity toward DNA compared to RNA, as evidenced by the higher fluorescence response (Figure 6). The most significant fluorescence increase upon binding to nucleic acids is observed for the complex dye **3b**-DNA, which shows a 145-fold increase in fluorescence intensity, and complex dye **3a**-poly(A)·poly(U), exhibiting a remarkable 256-fold increase (Table 5). The lowest fluorescence response is observed when dye 3a interacts with poly(A) (Figure 6c). This difference can be attributed to the fact that poly(A) is a single-stranded polynucleotide.

#### 2.2.5. Docking Studies

**Figure 7:**
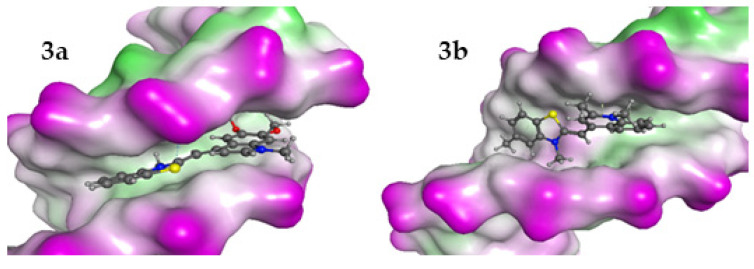
The best poses for the interaction complexes of the dyes **3a** and **3b** with dsDNA. The purple color represents the hydrophilic areas in DNA.

To explore the preferred modes of interaction of **3a** and **3b** with dsDNA and dsRNA, we conducted a docking study. This study explored several potential interaction mechanisms, including binding within the minor or major groove as well as intercalation. The docking of the ligands in both major and minor grooves of dsDNA is a kind of standard. In contrast, the intercalators are puzzling, as they are positioning themselves between the base pairs, and thus generating de novo holes in dsNA. The size of the hole depends on the dimensions of the intercalating ligands. To address this complexity, we used a crystallized dsDNA structure containing intercalated ligands of varying sizes as the basis for our docking studies. The preparation of dsRNA models was based on modification of the dsDNA structures. This approach enabled us to identify the most suitable binding pockets for the tested ligands while allowing for a comparison of interaction energies across different pocket dimensions. Induced fit docking and full minimization docking studies were performed. The interaction energies of dyes with dsDNA and dsRNA models are summarized in Table 6, and the minor groove binding interactions of **3a** and **3b** are illustrated in Figure 7.

The results indicated that for interaction with dsDNA, the preferred position for the ligand is between two A-T–A-T pairs, while in the dsRNA model, the dye **3a** prefers G-C–G-C pairs and **3b** prefers A-U–G-C. The interaction energies of ligands with dsDNA and dsRNA models indicate that the minor groove of dsDNA is the preferred binding site for all ligands. In both cases, the interaction between the ligands and dsDNA is energetically more favorable than with dsRNA. That is in general agreement with the determined binding constants K_b_.

#### 2.2.6. CD Spectroscopy

**Figure 8:**
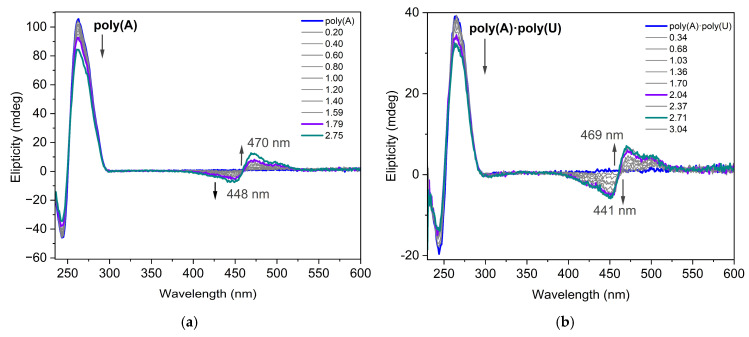
CD spectra of dye 3a interacting with (**a**) poly(A) (C = 8.7 × 10^−5^ mol/L) and (**b**) poly(A)·poly(U) (C = 5 × 10^−5^ mol/L in phosphate buffer 10 mM, pH 7.5) for different ratios r = [dye]/[polynucleotide].

Circular dichroism (CD) spectroscopy is an invaluable tool for studying the binding of small molecules to chiral macromolecules such as nucleic acids (NA). During the titration experiment, the nucleotide concentration was kept constant while the dye was gradually added to the NAs solution. The CD spectra of dye **3a** interacting with poly(A) and poly(A)·poly(U) are presented in Figure 8. Poly(A) is a single-stranded nucleic acid consisting entirely of adenine bases. The concentration of poly(A) in terms of nucleotides was determined using UV absorbance measurements at 260 nm, applying a molar extinction coefficient of 10,000 L·mol^−1^·cm^−1^ [31]. The CD spectrum of the poly(A) structure at pH 7.0 has an intense positive band around 264 nm and a negative band around 248 nm. Dye molecule itself does not show any characteristic CD in its absorption interval. The induced CD signal (ICD) observed in the absorption band of a nonchiral ligand is direct evidence of interaction with NA. In both cases shown in Figure 8, there is evidence of the dye interacting with the nucleotides as an aggregate, likely a dimer, according to the theoretical calculations. In the spectra of dye **3a** interacting with poly(A) and poly(A)·poly(U) exciton-coupled bisignate ICD bands are observed. The appearance of exciton-coupled bisignate ICD bands strongly support the aggregate binding along the polynucleotide.

The CD spectra measured along the titration of poly(A) with dye **3a** (Figure 8a) were analyzed with a multivariate data analysis method to gain insight into the potential existence of more than one interaction complex between the dye and poly(A) [32]. The method allowed the calculation of the concentration profiles and pure spectra for the considered components (Figure S18a,b). In this case, only two components were observed, which were related to the DNA and to the dye:DNA complex. However, in the case of data recorded along the titration of poly(A)·poly(U), an additional complex was observed at intermediate dye:DNA ratios (shown in red in Figure S18c,d). This is somewhat logical considering that the double stranded conformation is a scaffold more complex for dye docking than that of poly(A). According to the resolved CD spectrum of this intermediate, the double-stranded conformation of poly(A)·poly(U) is less affected by the interaction with the ligand than at higher dye/DNA ratios. Moreover, a weak induced CD signal appeared. Considering the limited structural information provided by CD, this species could be related to the interaction of the double-stranded poly(A)·poly(U) with one or two ligands.

## 3. Experimental Part

### 3.1. General

The starting chemicals and the solvents are commercial products HPLC grade and were used as supplied. The starting compounds **1a**, **1b**, **2a**, and **2b** were prepared according to previously described procedures [8]. The progress of the chemical reactions is followed by thin layer chromatography (TLC) ALUGRAM^®^ SIL G/UV 254-60 Macherey-Nagel, Düren, Germany, ready-to-use plates with a thickness of the silica layer of 0.2 mm. Melting points of the reaction products were measured on Boetius PHMK 0.5 apparatus and are uncorrected. NMR spectra (1H-, 13C-NMR) were obtained on a Bruker Avance II + NMR spectrometer operating at 500 MHz for 1H- and 125 MHz for 13C-NMR in DMSO-d6 as a solvent. The chemical shifts are given in ppm (δ) using tetrame-thylsilane (TMS) as an internal standard. Matrix-assisted laser desorption/ionization–time of flight (MALDI-TOF) analyses were performed using a Shimadzu Axima Confidense TM mass spectrometer (Kratos Analytical Ltd., Stratford, UK) equipped with ionization source of variable ion extracting energy (linear +25 kV/−20 kV, reflectron +20 kV/−20 kV) working of positive and negative ion operation, linear flight tube of 1.2 m drift length, and reflectron effective 2.0 m drift length and detectors: linear mode—multiple dynodes and reflection mode—fast micro-channel plate. All experiments shown here were made in the linear positive ion mode due to the increased sensitivity in this range analyzing relatively low molar masses of the investigated probes. Samples were prepared by dissolving dyes **3a** and **3b** in ethanol:DMSO = 1:1. No matrix or cationizing agent was used in the sample preparation for MALDI-TOF experiments. A small drop of the dye solution (5 mg/mL) was applied to the plate and after evaporation of the solvent it was introduced for analysis in the mass spectrometer. Ions were formed by laser desorption at 337 nm N2 laser (3 ns pulse width, 100 mJ per laser shot, 50 Hz maximum pulse rate) and accelerated with +25 kV (positive ion operation). All mass spectra shown were collected, baseline corrected, and smoothed by the Shimadzu Biotech Launchpad 2.9.9.3 software. Molar mass calculations were made by the data system using Kratos Analytical Polymer v.2.9 software. UV/Vis spectra were measured on Shimadzu UV-1800 spectrophotometer, and the fluorescence spectra were obtained on a PerkinElmer LS45 fluorescence spectrophotometer (slit widths is 5 nm). Dyes stock solutions were prepared in DMSO, and further diluted with TE-buffer (Tris-HCl 10 mM pH 8.0; EDTA 1 mM, pH 8.0). The absorption and emission properties of dyes **3a** and **3b** were investigated in TE buffer in the absence and in the presence of nucleic acids. CD spectra were recorded on a Jasco J-815 spectropolarimeter equipped with a Peltier accessory for temperature control.

### 3.2. Synthesis of the Asymmetric Monomethine Cyanine Dye 3a

A total of 0.29 g (0.001 mol) of 2-methylbenzo[*d*]thiazolium iodide **1a** and 0.44 g (0.0012 mol) 4-chloro-6,7-dimethoxy-1-methylquinolinium iodide **2a** were mixed and finely ground in a mortar. The mixture was transferred to a round bottom flask equipped with electromagnetic stirrer and rubber septum. The flask was flushed with argon and 5 mL ethanol and 5 mL dichloromethane were added by syringe. N,N-Diisopropylethylamine (DIPEA) 0.28 g (0.0022 mol, 0.38 mL) was added dropwise by syringe. The reaction mixture was stirred vigorously for 3 h at room temperature. The resulting precipitate was filtered off on a Buhner funnel, washed with three portions of ethanol (15 mL each) and purified by recrystallization from ethanol/dichloromethane. *(E)*-6,7-dimethoxy-1-methyl-4-((3-methylbenzo[d]thiazol-2(3H)-ylidene)methyl)quinolin-1-ium iodide **3a**. Yield: 0.28 g (57%) red needles. Mp > 250 °C. ^1^H-NMR (600 MHz, DMSO-d_6_, δ (ppm)): 3.94 s (3H, NCH_3_); 4.08 s (6H, OCH_3_); 4.22 s (3H, N^+^CH_3_); 6.66 s (1H, CH); 7.34 dd (1H, CH, J = 8 Hz); 7.36 s (1H, CH); 7.42 d (1H, CH, J = 8 Hz); 7.55 dd (1H, CH, J = 8 Hz); 7.68 d (1H, CH, J = 8 Hz); 7.79 s (1H, CH); 7.95 d (1H, CH, J = 8 Hz), 8.54 d (1H, CH, J = 8 Hz). ^13^C-NMR (DMSO-d_6_, δ (ppm)): 33.85, 43.41, 56.85, 57.04, 87.52, 99.55, 105.02, 108.45, 112.67, 119.51 (C), 123.10, 123.70 (C), 124.28, 128.35, 135.42 (C), 141.20 (C), 143.32 (C), 147.88 (C), 149.63 (C), 154.86 (C), 158,39 (C). ^13^C-DEPT-NMR (135 MHz, DMSO-d_6_, δ (ppm)): 33.85 (CH_3_), 43.41 (CH_3_), 56.85 (CH_3_), 57.04 (CH_3_), 87.52 (CH), 99.55 (CH), 105.02 (CH), 108.45 (CH), 112.67 (CH), 123.10 (CH), 124.28 (CH), 128.35 (CH). MALDI-TOF (*m*/*z*) Calculated Chemical Formula: C_21_H_21_N_2_O_2_S^+^; Calculated Exact Mass = 365.13; Found Mass [*m*/*z*]^+^ = 364.4613 (100%), 365.4578 (30%), 366.4557 (10%)

### 3.3. Synthesis of the Asymmetric Monomethine Cyanine Dye 3b

A total of 0.31 g (0.001 mol) of 2,5-dimethylbenzo[*d*]thiazolium iodide **1b**, 0.38 g (0.0012 mol) 4-chloro-6,7-dimethoxy-1-methylquinolinium iodide **2b** and N,N-Diisopropylethylamine (DIPEA) 0.28 g (0.0022 mol, 0.38 mL) were mixed and treated in the manner described above. The resulting precipitate was filtered off on a Buhner funnel, washed with ethanol (3 × 15 mL each portion) and recrystallized from ethanol/dichloromethane. *(E)*-4-((3,5-dimethylbenzo[d]thiazol-2(3H)-ylidene)methyl)-1,2-dimethylquinolin-1-ium iodide **3b**. Yield 0.31 g (67%) red powder. ^1^H-NMR (600 MHz, DMSO-d_6_, δ (ppm)): 2.45 s (3H, PhCH_3_); 2.84 s (3H, PhCH_3_); 4.02 s (3H, N^+^CH_3_); 4.20 s (3H, N^+^CH_3_); 6.77 s (1H, CH); 7.20 d (1H, CH, J = 8 Hz); 7.23 s (1H, CH); 7.52. s (1H, CH); 7.72 dd (1H, CH, J = 7.6 Hz); 7.84 d (1H, CH, J = 8 Hz); 7.96 dd (1H, CH, J = 8 Hz); 8.12 d (1H, CH, J = 9 Hz); 8.71 d (1H, CH, J = 8.3 Hz). ^13^C-NMR (DMSO-d_6_, δ (ppm)): 21.69, 23.23, 34.04, 37.46, 87.33, 110.84, 113.43 (C), 113.45, 118.66, 121.15 (C), 122.74, 125.77, 126.76, 133.50, 138.65 (C), 139.45, 141.10 (C), 148.16 (C), 154.40 C), 154.45 (C), 159.94 (C). ^13^C-DEPT-NMR (135 MHz, DMSO-d_6_, δ (ppm)): 21.70 (CH_3_), 23.22 (CH_3_), 34.05 (CH_3_), 37.47 (CH_3_), 87.35 (CH), 110.88 (CH), 113.47 (CH), 118.70 (CH), 122.78 (CH), 125.77 (CH), 125.80 (CH), 126.78 (CH), 133.52 (CH). Calculated Chemical Formula: C_21_H_21_N_2_S^+^; Calculated Exact Mass = 333.14; Found Mass [*m*/*z*]^+^ = 332.6123 (100%).

### 3.4. Docking

For the molecular docking study, a set of 3D X-ray diffraction (XRD) structures was selected and used as docking templates. The chosen PDB codes for dsDNA structures included 108D, 185D, 1DNE, 1XRW, 1Z3F, and 2DES. All complexes represented dsDNA with an intercalated ligand, with the only exception being 1DNE. For all XRD structures with an intercalated ligand, the original ligand position in the crystal was used as a reference to guide the new ligand placement. The 1DNE structure served as a control to explore potential complex formation along the DNA grooves. The netropsin ligand was removed from 1DNE and the Edelsbrunner method [33], as implemented in the MOE software [34], was used to define centers along both the minor and major grooves. These defined centers were then used to position ligands within the grooves during docking.

The Induced Fit methodology was employed for ligand docking since intercalation creates a cavity within dsDNA that optimally accommodates the intercalating molecule. To prepare the XRD structures for docking and simulate their behavior in solution, we conducted short molecular dynamics (MD) simulations (50 ns) using NAMD 2.14 software. The AMBER12EHT force field was applied, with explicit water molecules and periodic boundary conditions at 310 K for each XRD structure. For dsRNA models, we modified the corresponding dsDNA XRD structures to generate dsRNA constructs, followed by a 250 ns molecular dynamics simulation using the same conditions as for dsDNA. Given that our ligands can interact with nucleic acids either through intercalation or by binding within the minor or major grooves, both interaction modes were analyzed. To prevent ligand interactions with nucleic acid termini, the 1XRW model was extended to eight base pairs (bp). Additionally, independent dsDNA and dsRNA models (not derived from XRD structures) were generated, each consisting of 12 bp, to ensure that all ligand regions were sufficiently distant from the nucleic acid ends. This model produced the most reliable results for ligand–groove interactions. During the initial docking phase, ligand conformations were positioned using the Triangle Matcher algorithm, allowing for up to 1,000,000 poses within cavities. Additionally, poses obtained with the AlphaPMI algorithm were added to the pool of poses. The generated poses were then ranked using the London dG scoring function, and the top 100 unique poses were selected for further minimization using the AMBER12EHT force field and the Generalized Born solvent model. During this minimization, dsNA atoms located more than 6 Å away from the ligand, as well as backbone atoms, were treated as immovable. Final pose evaluation was performed using the GBVI/WSA dG scoring method [35]. In a previous induced-fit docking study, the fixed nucleic acid backbone led to suboptimal ligand interactions due to the unchanged cavity parameters. We performed an additional minimization step for the top 10 ligand–nucleic acid complexes from the previous docking results to overcome this limitation. This step employed the AMBER12EHT force field and the Generalized Born solvation model without any constraints. The final ligand positions and their interaction energies with dsNA were computed using the GBVI/WSA scoring function.

The ligand preparation for docking involved appropriate protonation guided by experimental data, followed by a conformational search. Conformations were generated using Low Mode Molecular Dynamics with the AMBER12EHT force field and the generalized Born solvent model. Only those within 10 kcal/mol of the lowest-energy conformation were selected for docking.

## 4. Conclusions

Two new asymmetric monomethine cyanine dyes, containing hydrophobic functional groups attached to the chromophore part of their molecules, have been synthesized. These modifications enhance their affinity for the hydrophobic regions of nucleic acids within the biological matrix. The chemical structures of these dyes were determined using ^1^H-NMR, ^13^C-NMR, ^13^C-DEPT 135 NMR, and MALDI-TOF mass spectrometry. Due to the existing difficulties hindering the MALDI-TOF mass spectrometric analysis of cationic dyes, a novel methodology was developed and successfully applied to study the new dyes in MALDI-TOF mass experiments without requiring a matrix or cationizing compounds. The photophysical properties of the dyes were investigated both experimentally and theoretically. The binding affinity and interaction modes of the dyes with nucleic acids (NAs) were evaluated through UV/Vis titration, fluorescence titration, circular dichroism, and molecular docking. The results indicate that the dyes exhibit greater sensitivity toward DNA than RNA. The most significant fluorescence enhancement upon binding to nucleic acids was observed for the complex of dye **3a** with poly(A)·poly(U), demonstrating a remarkable 256-fold fluorescence increase. Similarly, the complex of dye **3b** with dsDNA showed a 145-fold increase in fluorescence. In contrast, the lowest fluorescence response was observed when dye **3a** interacted with the single-stranded polynucleotide poly(A). Both experimental and theoretical data confirm that the dyes exhibit a strong tendency to aggregate. Using DFT and TDDFT methods, the molecular geometry of the dyes and their potential to form various dimers in solution were characterized. The energy difference between the orbitals involved in the absorption transition is higher for the H2 dimer than for the monomer, which explains the hypsochromic shift in the absorption maxima for dimer aggregates. Fluorescence titration experiments and CD spectroscopy revealed that the dyes, upon binding to nucleic acids, undergo changes in their spectra with pronounced bioaggregachromism—spectral changes upon formation of aggregates on the surface of the respective biopolymers. The new dyes are highly suitable for application as fluorogenic probes in biomolecular recognition techniques.

## Figures and Tables

**Table 1:** The M062X/6-31G(d,p) computed relative enthalpy ΔH and Gibbs free energy differences ΔG (with respect to the most stable conformer), as well as the predicted absorption maxima and their oscillator strength f (from HFS/6-31+G(d,p) computations) for the three conformers of **3a** and **3b**.

Isomer	∆H [kcal/mol]	∆G [kcal/mol]	λ _abs_ [nm]	f
**3a** s-trans	0.0	0.0	496	0.8334
**3a** s-cis	2.5	3.4	494	0.8573
**3a** trans out	2.5	3.1	528	0.4577
**3b** s-trans	0.0	0.0	507	0.7145
**3b** s-cis	1.9	3.6	502	0.7255
**3b** trans out	3.3	4.2	557	0.4286

**Table 2:** M062X/6-31G(d,p) calculated NBO, Hirshfeld atomic charges, and EPN values for the nitrogen atoms of **3a** and **3b** molecules.

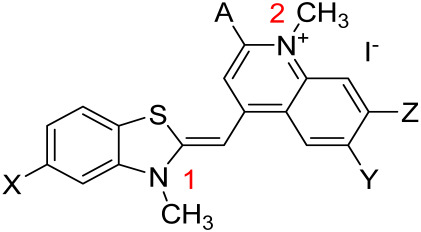					
			q_NBO_	q_Hirsh_	EPN
**3a**	s-trans	N1	−0.3890	−0.0055	−18.1593
		N2	−0.3353	0.0312	−18.1243
	s-cis	N1	−0.3969	−0.0022	−18.1504
		N2	−0.3390	0.0285	−18.1213
	trans out	N1	−0.3968	−0.0109	−18.1659
		N2	−0.3306	0.0349	−18.1175
**3b**	s-trans	N1	−0.3825	−0.0012	−18.1559
		N2	−0.3449	0.02281	−18.1316
	s-cis	N1	−0.3867	0.0045	−18.1435
		N2	−0.3503	0.0190	−18.1303
	trans out	N1	−0.3879	−0.0050	−18.1617
		N2	−0.3411	0.0257	−18.1259

**Table 3:** The relative energies and the calculated Boltzmann distributions for each of the eight studied dimers of dye **3a**.

Dimer	ΔG [kcal/mol]	Distribution, %
H1	4.58	0.04
H2	0.00	89.75
H3	2.70	0.94
H4	1.35	9.27
J1	10.56	0.00
J3	5.59	0.01
J4	7.74	0.00

**Table 4:** PCM/ HFS/6-31+G(d,p) calculated and experimental absorption maxima (nm) for the monomeric dyes and the most stable π-stacked dimers for dyes **3a** and **3b**.

Dye	Monomer—TE Buffer		Monomer *—DMSO	Dimer TE buffer	
	λ_calc_ [nm]	λ_exp_ [nm]	λ_exp_ [nm]	λ_calc_ [nm]	λ_exp_ [nm]
**3a**	496 (0.8334)	490	498	479 (1.343)	464
**3b**	507 (0.7145)	498	507	484 (1.104)	478

* The dyes do not aggregate in DMSO.

**Table 5:** Fluorescent properties of the dyes and their complexes with nucleic acids. I_0_ values refer to the fluorescence of the dyes neat in TE buffer in concentrations given in Figure 3. The fluorescence of the complexes (Imax) refers to the highest NA concentration, as presented in Figure 6.

Dye	NA	λ_ex_ (nm)	$\lambda_{em}^{dye}$(nm)	$\lambda_{em}^{complex}(\mathrm{nm})$	*I*_0_ (a.u.)	I_max_ (a.u.)	*I*/*I*_0_	K_b_ × 10^6^	Q	n
**3a**	DNA	495	531	531	9.5	622.1	65.5	8.0	78.6	0.08
	RNA	504	526	525	4.9	136.7	27.9	2.8	38.0	0.22
	poly(A)	490	592	592	3.3	35.8	10.8	9.5	11.5	0.89
	poly(A)·poly(U)	501	531	531	3.4	967.1	282.0			
**3b**	DNA	497	580	536	2.3	337.3	145.4	1.5	173.6	0.41
	RNA	476	619	538	2.5	217.4	87.0	2.6	111.8	0.11

**Table 6:** Interaction energies (ΔG in kcal/mol) of ligands with dsDNA and dsRNA models. Bold numbers indicate the most favorable interaction energies.

Ligand	dsDNA		dsRNA	
	intercalation	minor groove binding	intercalation	minor groove binding
	ΔG			
**3a**	−10.7	**−13.5**	−10.6	−9.5
**3b**	−8.9	**−14.2**	−9.2	−9.0

## Data Availability

The original contributions presented in this study are included in the article/Supplementary Materials. Further inquiries can be directed to the corresponding authors.

## Supplementary Materials

The supporting information can be downloaded at: https://www.mdpi.com/article/10.3390/bios15030187/s1. Figure S1. ^1^H-NMR spectra of dye **3a** in DMSO-d_6_. Figure S2. ^1^H-NMR spectra of dye **3a** in DMSO-d_6_ as a solvent in the range 3.60–4.50 ppm. Figure S3. ^1^H-NMR spectra of dye **3a** in DMSO-d_6_ in the range 6.40–8.80 ppm. Figure S4. Full range HSQC spectra of dye **3b** in solvent DMSO-d_6_. Figure S5. (a–d) ^1^H-NMR spectra of dye **3a** in DMSO-d_6_. (e) ^13^C-DEPT-NMR in DMSO-d_6_ of dye **3a**; (f) HSQC spectra of dye **3a** in DMSO-d_6_. Figure S6. ^13^C-NMR spectra in DMSO-d_6_ of dye **3a** in the full range. Figure S7. HSQC spectra in DMSO-d_6_ as a solvent of dye **3a** in the full range. Figure S8. Full range ^1^H-NMR spectra in DMSO-d_6_ of dye **3b**. Figure S9. ^1^H-NMR spectra of dye **3b** in DMSO-d_6_ in the range 2.00–4.50 ppm. Figure S10. ^1^H-NMR spectra of dye **3b** in DMSO-d_6_ in the range 5.80–9.00 ppm. Figure S11. Full range ^13^C-NMR spectra of dye **3b** in solvent DMSO-d_6_. Figure S12. Full range ^13^C-DEPT-NMR spectra of dye **3b** in solvent DMSO-d_6_. Figure S13. MALDI-TOF mass spectrum of dye **3a**. Figure S14. MALDI-TOF mass spectrum of dye **3b**. Figure S15. Chemical structure, calculated chemical formula, exact theoretical mass, and MALDI-TOF mass spectra for the molecular peak of dyes **3a** and **3b**. Figure S16. M062X/6-31G(d,p) optimized molecular structures of **3a** H-type trimer in water medium: left-side view, right-top view. Figure S17. Molecular orbitals involved in the S0→S1 transition for the monomer, dimer and trimer of **3a** dye computed at M062X/6-31G(d,p) in water medium. Figure S18. Results of the application of Multivariate Curve Resolution based on Alternating Least Squares to the CD data set recorded along the titrations of poly(A) and poly(A)·poly(U) with dye **3a**.
