# Supplementary material for: Bioaggregachromism of Asymmetric Monomethine Cyanine Dyes as Noncovalent Binders for Nucleic Acids
Source: Biosensors (Basel). 2025 Mar 14;15(3):187. doi: 10.3390/bios15030187 (PMC11940764; doi:10.3390/bios15030187)
Supplement: Supplementary file 1 [file biosensors-15-00187-s001.zip › biosensors-3478545-supplementary.pdf]

Electronic Supporting Information

Bioaggregachromism of asymmetric monomethine cyanine dyes as noncovalent binders for nucleic acids

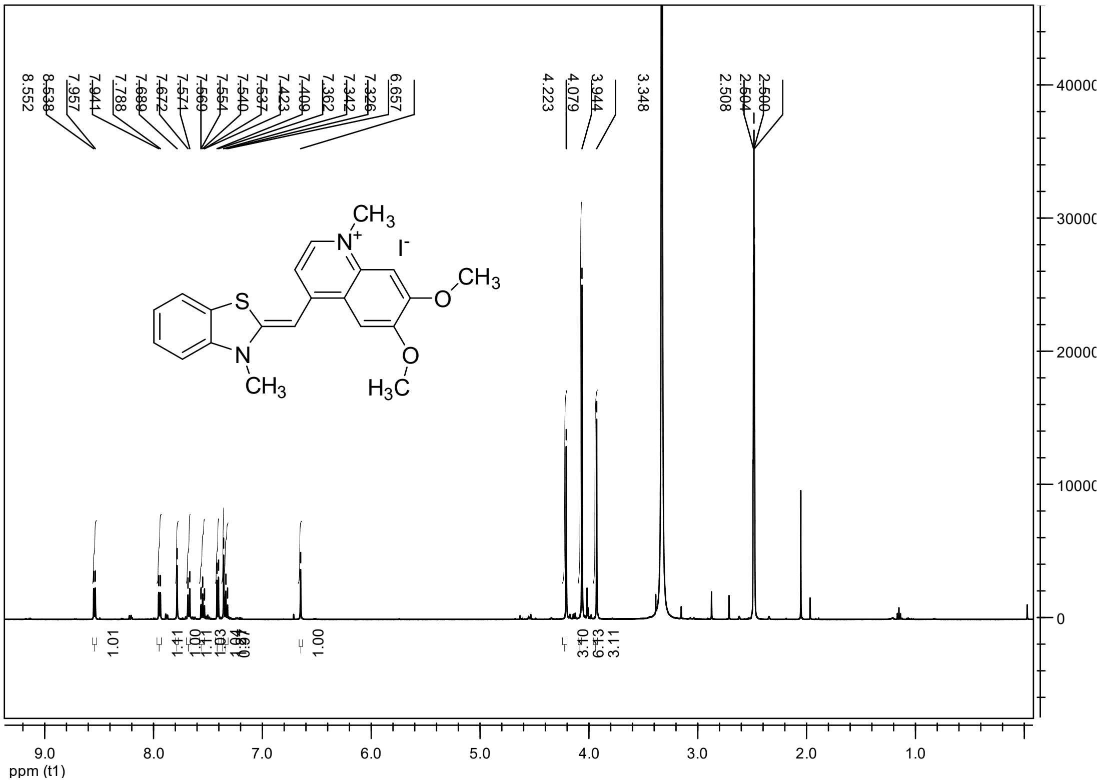

Figure S1. ^{1}H-NMR spectra of dye **3a** in DMSO-d_6.

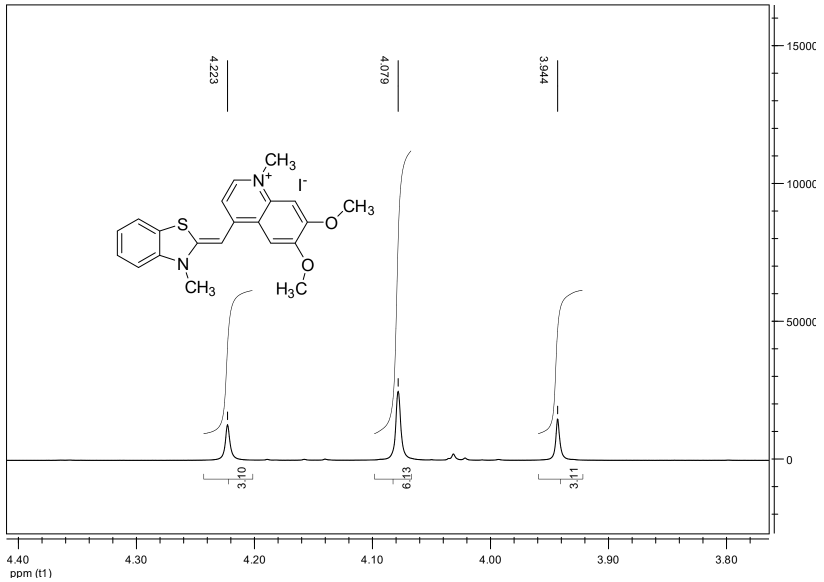

Figure S2. ^{1}H-NMR spectra of dye **3a** in DMSO-d6 as a solvent in the range 3.60-4.50 ppm.

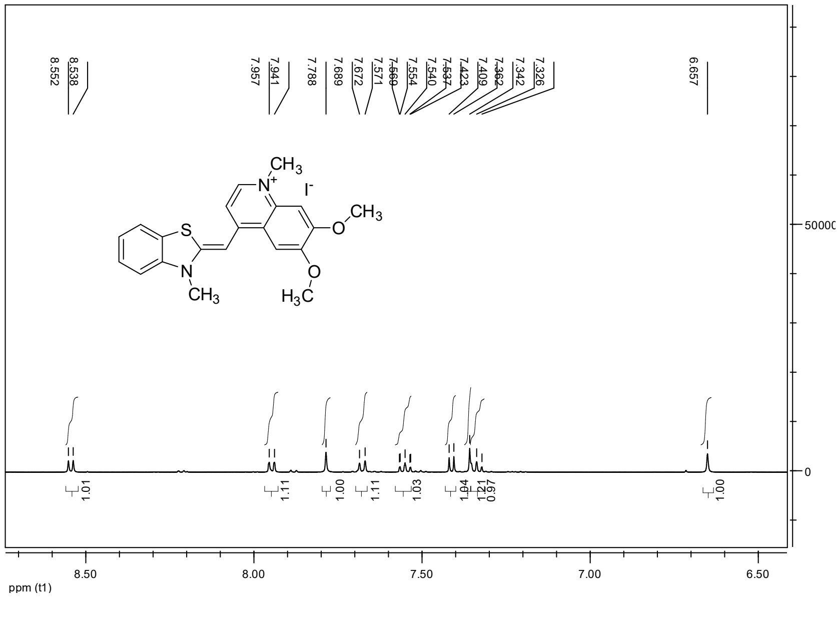

Figure S3. ^{1}H-NMR spectra of dye **3a** in DMSO-d6 in the range 6.40-8.80 ppm.

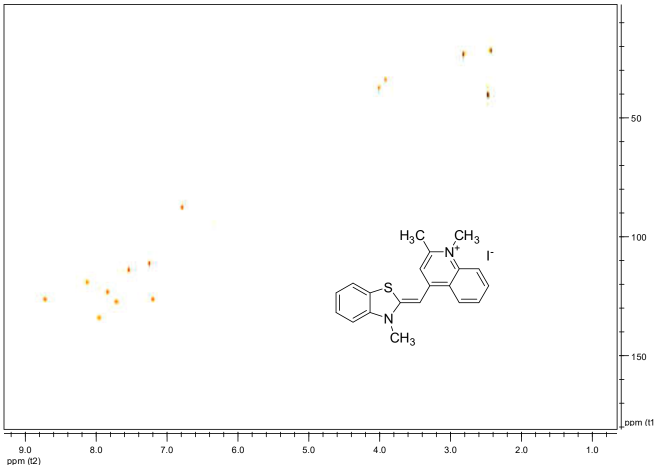

Figure S4. Full range HSQC spectra of dye **3b** in solvent DMSO-d_6.

a)

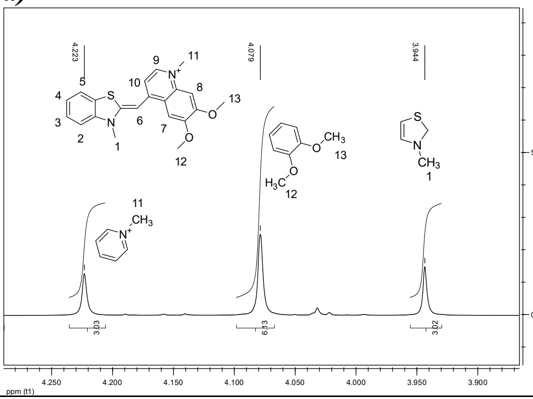

b)

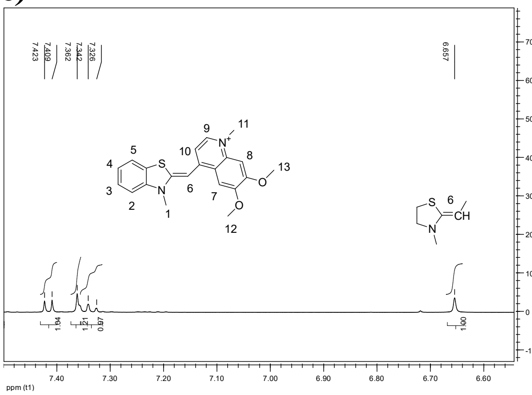

c)

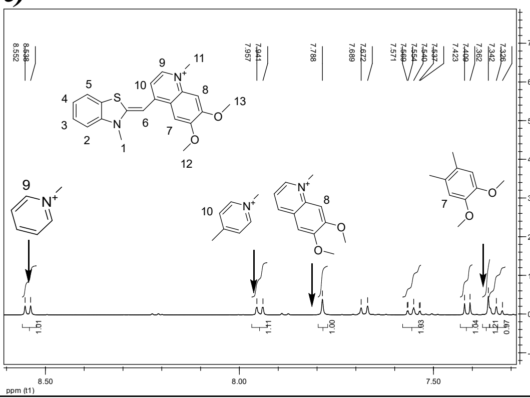

d)

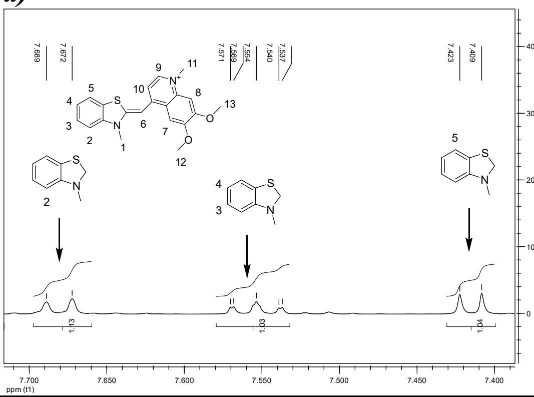

e)

f)

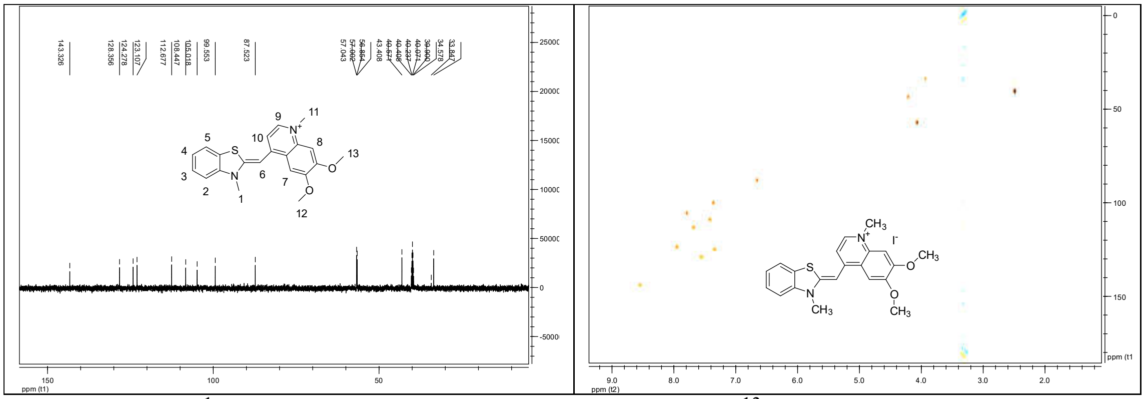

Figure S5. ***a-d)*** ^{1}H-NMR spectra of dye **3a** in DMSO-d$_6$; ***e)*** ^{13}C-DEPT-NMR in DMSO-d$_6$ of dye **3a**; ***f)*** HSQC spectra of dye 3a in DMSO-d$_6$.

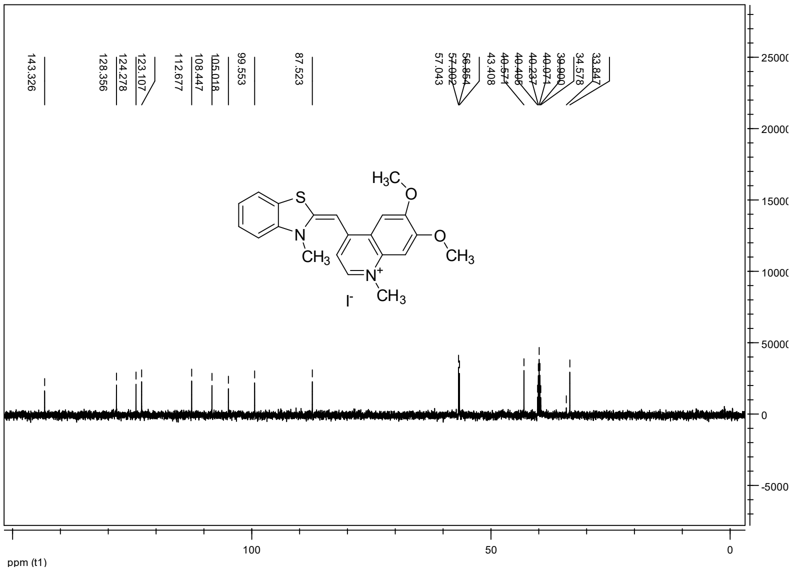

Figure S6. ^{13}C-NMR spectra in DMSO-d$_6$ of dye **3a** in the full range.

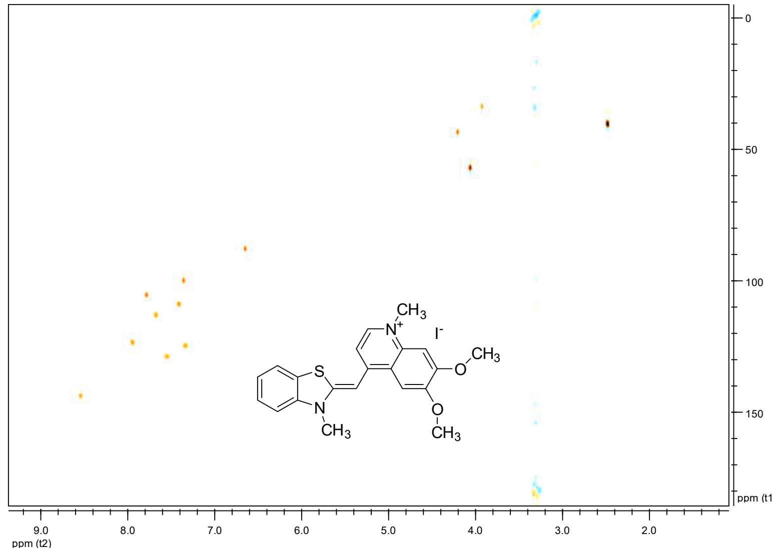

Figure S7. HSQC spectra in DMSO-d$_6$ as a solvent of dye **3a** in the full range.

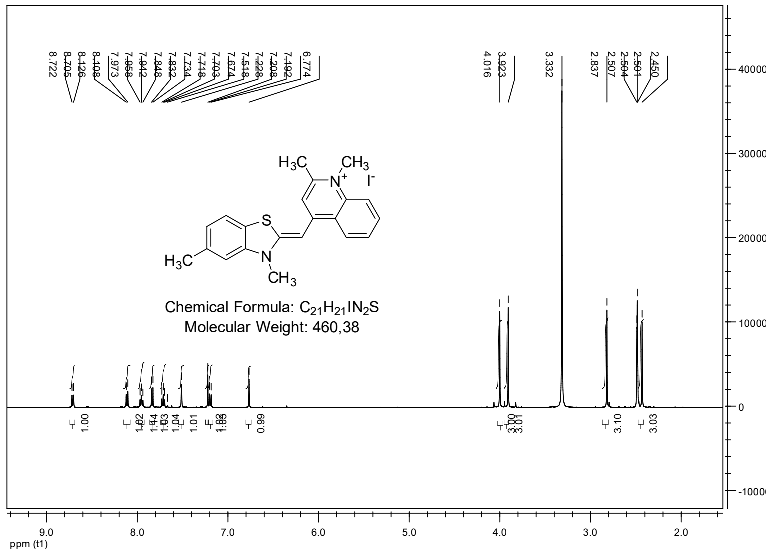

Figure S8. Full range ^{1}H-NMR spectra in DMSO-d$_6$ of dye **3b**.

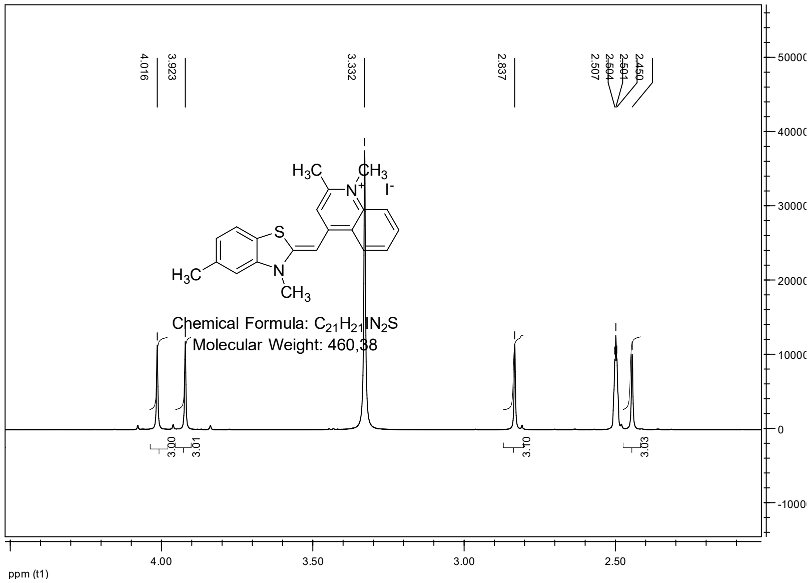

Figure S9. ^{1}H-NMR spectra of dye **3b** in DMSO-d$_6$ in the range 2.00-4.50 ppm.

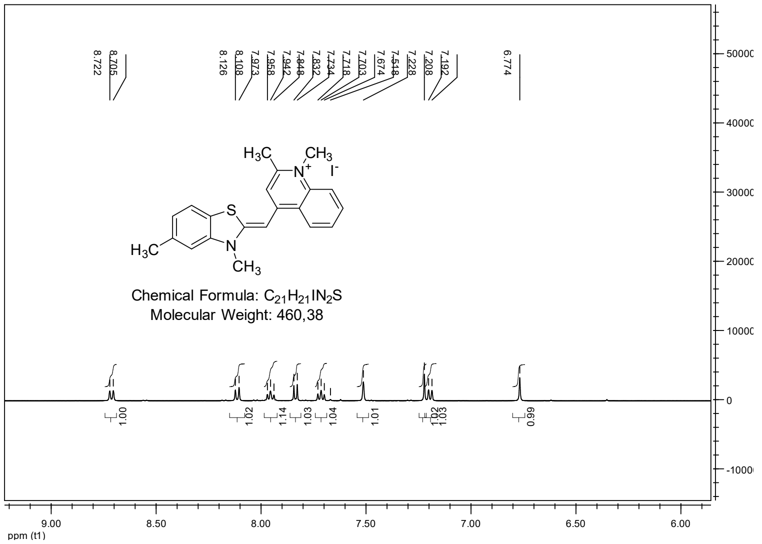

Figure S10. ^{1}H-NMR spectra of dye **3b** in DMSO-d$_6$ in the range 5.80-9.00 ppm.

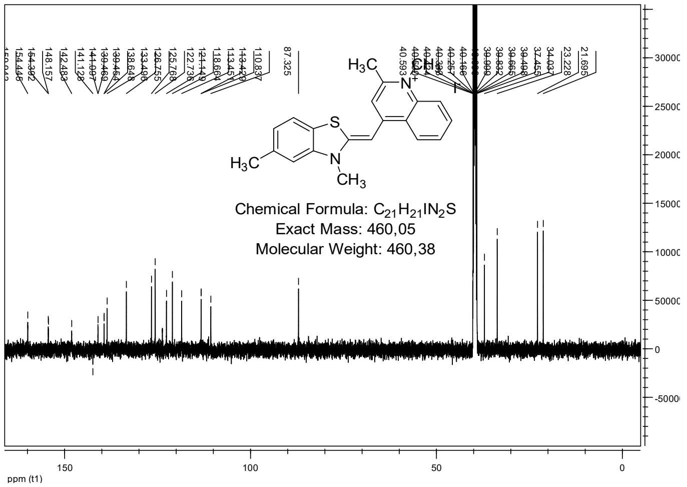

Figure S11. Full range ^{13}C-NMR spectra of dye **3b** in solvent DMSO-d_6.

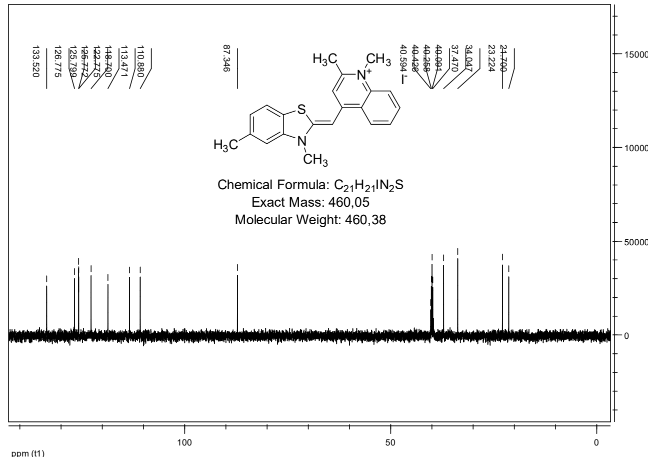

Figure S12. Full range ^{13}C-DEPT-NMR spectra of dye **3b** in solvent DMSO-d_6.

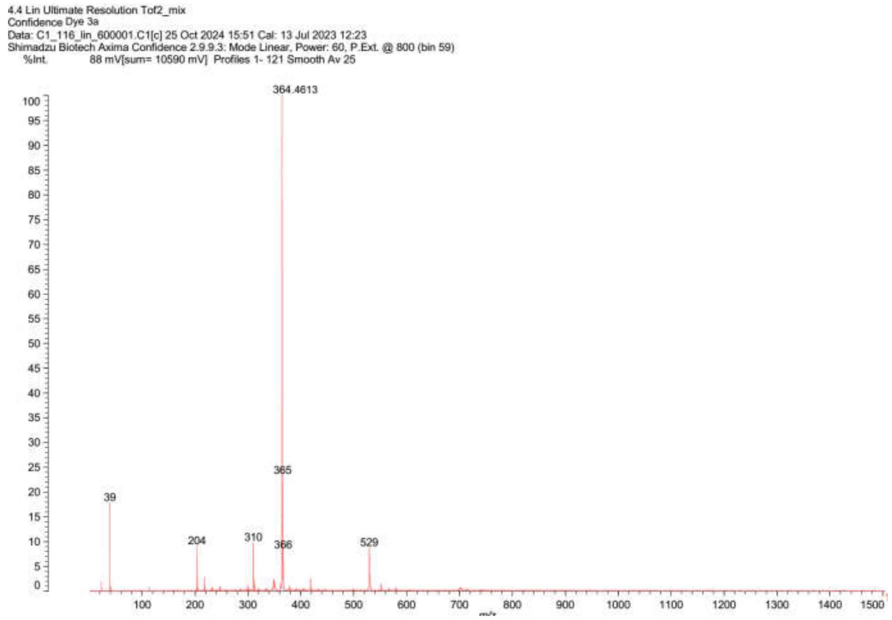

Figure S13. MALDI-TOF mass spectrum of dye **3a**.

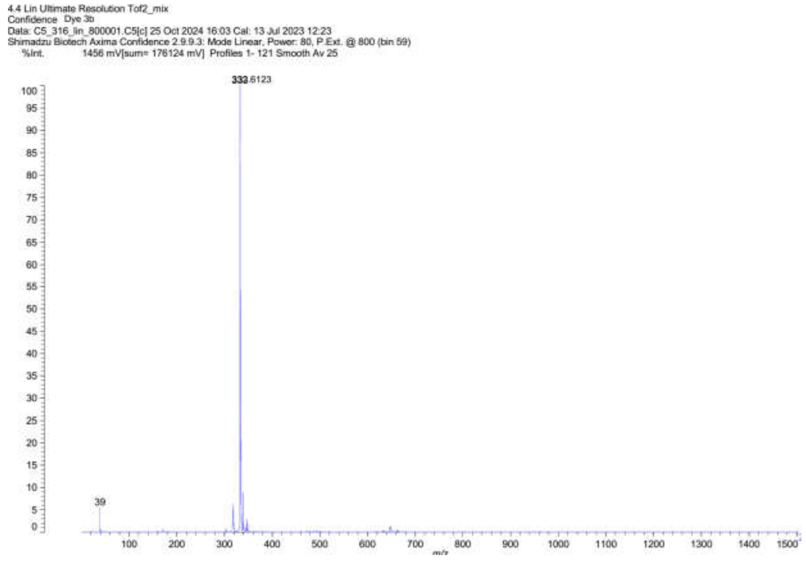

Figure S14. MALDI-TOF mass spectrum of dye **3b**.

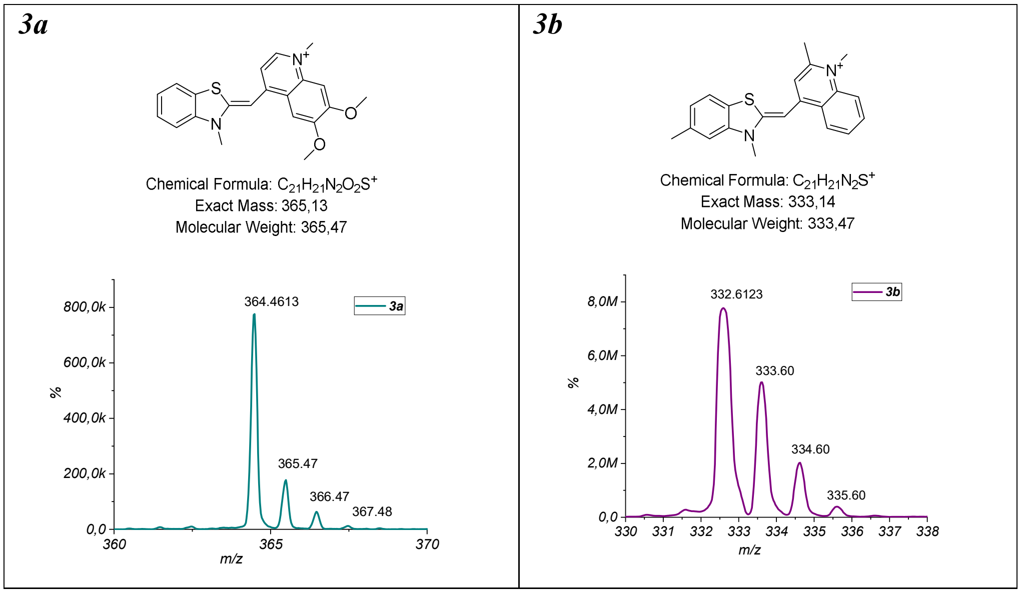

Figure S15. Chemical structure, calculated chemical formula, exact theoretical mass, and MALDI-TOF mass spectra for the molecular peak of dyes **3a** and **3b**.

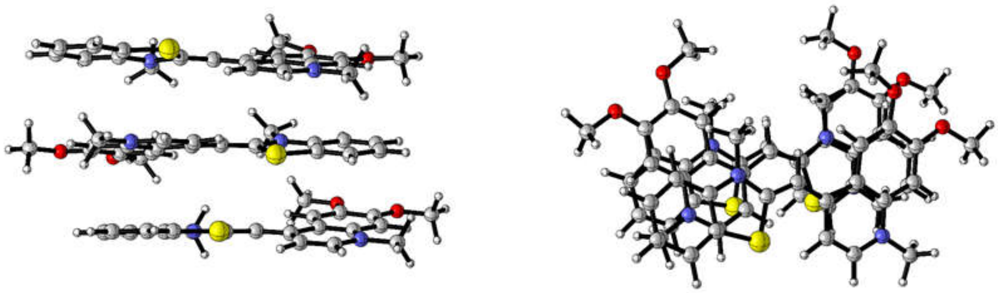

Figure S16. M062X/6-31G(d,p) optimized molecular structures of **3a** H-type trimer in water medium: left – side view, right–top view.

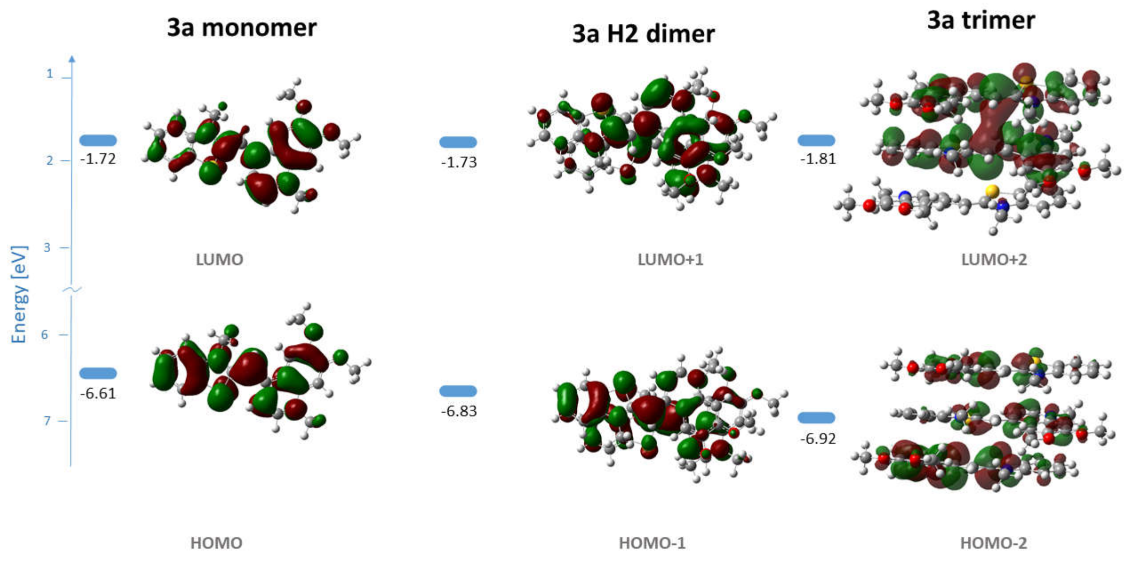

Figure S17. Molecular orbitals involved in the $S_0 \rightarrow S_1$ transition for the monomer, dimer and trimer of **3a** dye computed at M062X/6-31G(d,p) in water medium.

Calculated concentration profiles (a) and pure CD spectra (b) from the analysis of spectra shown in Figure 8a.

In this case, two components were needed to explain the experimental CD data (lack of fit was 1.9%).

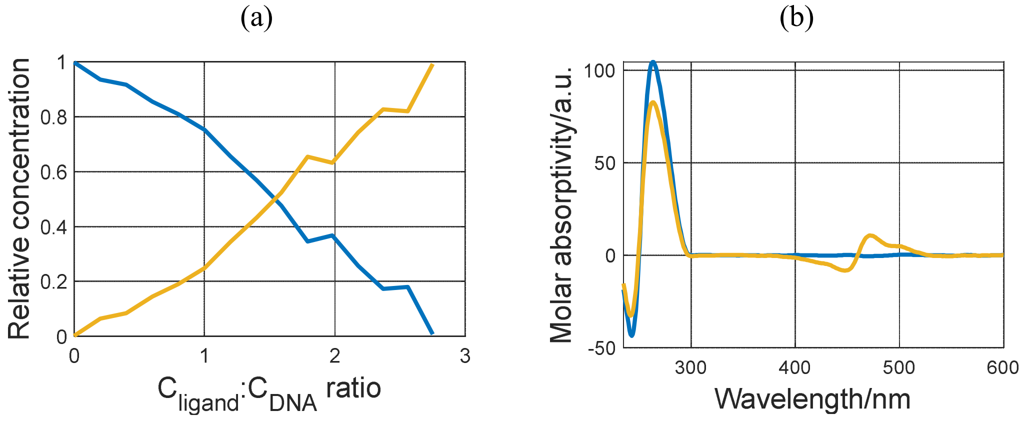

Calculated concentration profiles (c) and pure CD spectra (d) from the analysis of spectra shown in Figure 8b.

In this case, three components were needed to explain the experimental CD data (lack of fit was 1.8%).

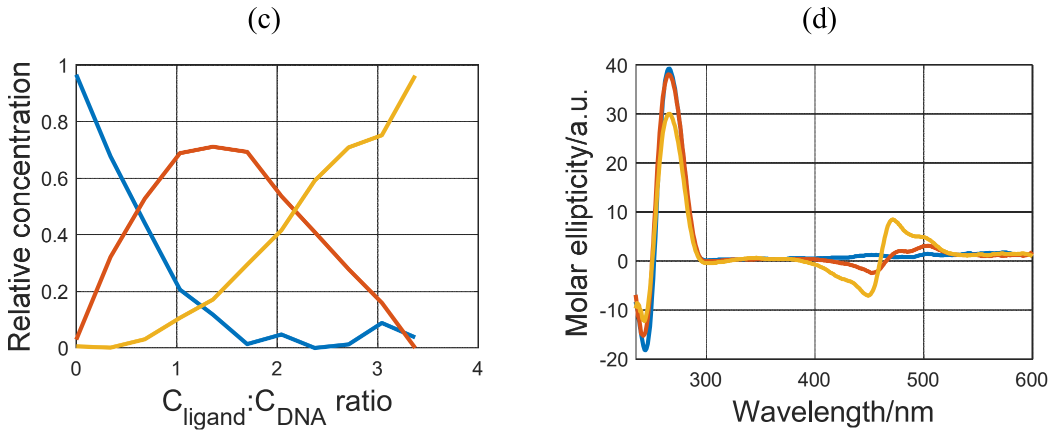

Figure S18. Results of the application of Multivariate Curve Resolution based on Alternating Least Squares to the CD data set recorded along the titrations of poly(A) and poly(A)·poly(U) with dye **3a**.
